# Sports participation and lifestyle in middle-aged adults with congenital heart disease

**DOI:** 10.1016/j.ijcchd.2024.100512

**Published:** 2024-04-05

**Authors:** C. Pelosi, R.M. Kauling, J.A.A.E. Cuypers, E.M.W.J. Utens, A.E. van den Bosch, W.A. Helbing, J.S. Legerstee, J.W. Roos-Hesselink

**Affiliations:** a Department of Cardiology, Erasmus MC, Rotterdam, the Netherlands; b Academic Center for Child and Adolescent Psychiatry Levvel/Amsterdam UMC/ University of Amsterdam, the Netherlands; c Department of Child and Adolescent Psychiatry/Psychology, Erasmus Medical Center-Sophia Children's Hospital, Rotterdam, the Netherlands; d Department of Pediatrics, Division of Cardiology, Erasmus University Medical Center, Sophia Children's Hospital, Rotterdam, the Netherlands

**Keywords:** Congenital heart disease, Sports, Long-term follow-up, Physical activity

## Abstract

**Background:**

Sports are associated with numerous physiological and psychological benefits. However, it is unclear to what extent adults with congenital heart disease(CHD) participate in sports and whether this is safe. Furthermore, little is known about lifestyle habits in this group.

**Methods:**

Consecutive patients diagnosed with atrial septal defect, ventricular septal defect, pulmonary stenosis, tetralogy of Fallot and transposition of the great arteries who underwent open-heart surgery during childhood between 1968 and 1980 were included in a longitudinal follow-up study. Patients underwent cardiological investigations every 10 years and completed a questionnaire regarding sports participation in 2011 and in 2021.

**Results:**

Of the 2021 cohort(n = 204, mean age:50 years, 46%female), 49% participated in sports. Compared to the general Dutch population they invested less time in sport(female: p = 0.03, male: p = 0.03). Patients participating in sports had better exercise capacity (p < 0.001) and lower average heart rate(p < 0.001). Patients engaged in sports reported better physical and mental health when compared to the norm and non-sporters. Mortality and cardiac events did not differ significantly between the 2 groups. Finally, no difference in lifestyle was found between adults with CHD and the general population, only smoking was less often reported among adults with CHD(p = 0.036).

**Conclusions:**

Adults with CHD are significantly less involved in sports than their peers. Patients who were engaged in sports had better physical and mental health compared to those who were not. Sport participation was associated with lower heart rate and better exercise capacity. No negative effect in terms of cardiac events was observed in sporters. No signs of bad lifestyle were observed in adults with CHD.

## 1 Introduction

Since the first surgical repair of a congenital heart defect in children in the 50s, every area of medical care of patients with a congenital heart disease (CHD) dramatically improved and, nowadays, over 97% of patients reach adulthood [1].

Sports participation is an important topic in current times and a healthy lifestyle is known to have an impact, not only on cardiovascular health, but also on the incidence of other medical problems (such as dementia, hip- and knee problems, cancer) and on mental health [2]. Recently, the European Society of Cardiology (ESC) provided guidelines encouraging a more active lifestyle and participation in sports, also in adolescences and adults with CHD. However, due to a lack of robust evidence, guidance and recommendations were largely based on experts’ opinions and are based on low level of evidence [3,4]. Further, a position paper from the ESC working group on adults with CHD and the ESC working group on sports discussed competitive sports participation in patients with CHD more in detail, proposing a step-by-step approach to assess suitability for (competitive) sports participation in these patients. These papers clearly stated that currently, data are limited, and more studies are warranted. Indeed, many physicians may still find it challenging and they encounter difficulties in advising their patients. The main concern is the assumed possible increased risk of adverse events related to participation in sports activities, such as sudden cardiac death or deterioration of cardiac function [5]. Therefore, adults with CHD may be advised against sports and/or possibly unnecessary restrictions were imposed during childhood resulting in lower participation in sports at older age [6,7]. However, interventional studies on children and adolescences showed positive impact and improved exercise capacity in CHD patients who were physically active [8,9]. Therefore, we investigated sports participation in adults with CHD. A previous study on this same cohort of patients showed less involvement in sports than the general Dutch population [6]. The aims of the current study were to assess sports participation in middle-aged CHD patients, describe trends over time, investigate associations with sports participation and to describe the present lifestyle habits of CHD patients compared to the general Dutch population.

## 2 Methods

### 2.1 Inclusion criteria, patient sample

Patients who underwent cardiothoracic surgery at young age (<15 years) for atrial septal defect (ASD), ventricular septal defect (VSD), pulmonary stenosis (PS), tetralogy of Fallot (ToF) or transposition of the great arteries (TGA) between 1968 and 1980 at Erasmus MC were included in the study. This is the 4th follow-up study (age>40 years old, in 2021) of the same cohort of patients already investigated in 1991, 2001 and 2011 [6,[10], [11], [12]]. Sports participation was assessed for the first time in 2011 and now again in 2021.

All the 343 patients who were alive in 2021, traceable and had previously participated in at least two follow-up studies, were invited to the current study. Of this group, 204 (59%) adults completed the sports questionnaire and 203 (59%) adults the lifestyle questionnaire. (table 1S - supplemetary material) For comparison over time, data of patients participating in both follow-ups concerning sportsparticipation of 2011 and 2021 were used (n = 174).

Patients were divided in two groups according to their CHD severity: mild (n = 141) (ASD, VSD and PS) and moderate/severe (n = 63) (ToF and TGA) [13].

### 2.2 Assessment procedure

The research protocol followed the ethical guidelines of the 1975 Declaration of Helsinki and was approved by the local ethical committee (MEC-2019 0465). Patients were not involved in the design of the study. All patients were approached uniformly, and provided written informed consent before participating in the study. In the outpatient clinic, a cardiologist performed cardiac and medical examinations. Before their visit to the hospital, a questionnaire was sent to the patients via a secured website (GemsTracker, Copyright©2011, Erasmus MC and Equipe Healthcare companies), to be completed at home one week before the visit. Due to technical issues or to patients’ personal reasons/preferences, 62 patients completed the paper version of the questionnaire during their hospital visit.

### 2.3 Instruments

1. Sports participation

Sports participation was assessed using the same questionnaire as in 2011. It included 4 items of the Baecke questionnaire, used to assess habitual physical activity [14]. Sports participation was defined as any type of physical activity in a group or alone, excluding daily cycling and walking. We classified the physiologic type of sports according to the ESC classification of 2021 [15]. According to this classification, sports can be divided into “skill”, “power”, “mixed”, and “endurance” sports [15]. For each category, the intensity was qualified as low, medium, or high. To compare the amount of sports participation of our cohort with the normal Dutch population per gender, we used the following categories, provided by the Dutch Central Institute of Statistics (CBS) (data of 2008): “Extensive” (5 or more hours of sports per week), “Little/Moderate” (1–4 h sports per week) and “Limited/None” (less than 1 h of sports per week) [16].

2. Clinical events:

Clinical events were identified as surgical or *trans*-catheter (re-)intervention, implantation of an implantable cardiac defibrillator (ICD) or pacemaker (PM), symptomatic heart failure, symptomatic arrhythmias, stroke, and death. Arrhythmias were deemed clinically relevant when medication, cardioversion, ablation, or hospitalization were needed. Heart failure was deemed significant if a patients was hospitalized or medication was started.

3. Exercise capacity

Maximal exercise capacity and peak oxygen consumption (VO_2_ max) were assessed with a bicycle ergometry test and presented as a percentage of the target in healthy adults of comparable age, gender, and height. A 20 W increase per minute protocol was used. All patients invited to participate to the study underwent the exercise test on the day of the visit.

4. Self-perceived quality of life

The self-perceived quality of life was assessed with the Linear Analogue Scale (LAS) [17]. Furthermore, the Short Form Health Survey (SF-36) was used for assessing quality of life [18].

5. Lifestyle

Lifestyle was assessed with the Rotterdam Questionnaire of Health Habits (Vragenlijst voor Gezondheidsgedragingen, ©2010E.M.W.J. Utens, K. Dulfer). Questions investigated alcohol use, smoking and drug use habits as well as dentist visits and fruit and vegetables consumption. When available, data were compared to the normal Dutch population. Normative data were derived from the CBS (data of 2020), Dutch Ministry of Public Health, Wellbeing and Sports (data of 2016) and Trimbos (Netherlands Institute of Mental Health and Addiction) (data of 2018) [19].

### 2.4 Statistical analysis

Categorical data were presented as percentages (frequency), whereas continuous data were presented as mean ± standard deviation. In case of skewed distribution, data were presented as median [25th-75th percentile]. Differences between categorical data were tested with the χ^2^ test. Longitudinal comparisons were assessed with the Stuart Maxwell test. The *t*-test, or if necessary, the Mann-Whitney-U test were used to define differences between continuous variables. Comparison between the scores on the SF-36 of the patients and the normal Dutch population were analysed with the one-sample *t*-test. Repeated measurements ANOVA and the McNemar test were used to analyse the differences over time of, respectively continuous and categorical, dependent variables.

Event-free survival in patients who participated in sports in 2011 versus those who did not is displayed as a Kaplan-Meier plot. The Peto and Peto test was used to compare the differences between these two groups. The patients that participated both in 2011 and 2021 (n = 174) or died between 2011 and 2021 (n = 10) were included in the mortality analysis.

Statistical significance was set at 0.05. Data were analysed with IBM SPSS Statistical Software v.25 and R Studio v. 4.1.0 for Windows.

## 3 Results

### 3.1 Sports participation

**Fig. 1:**
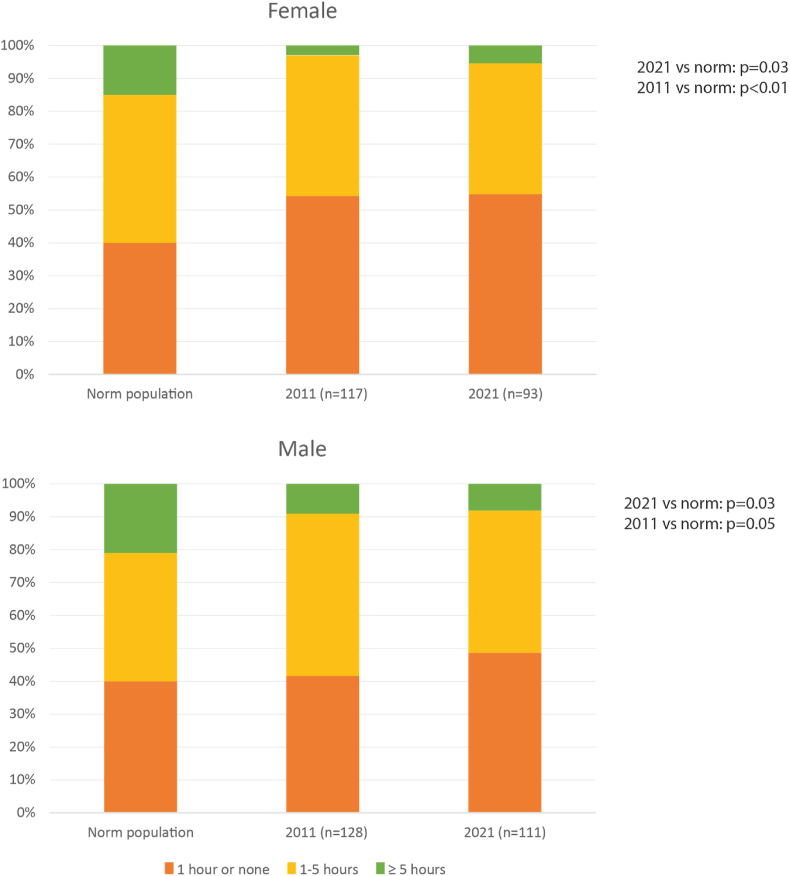
Sport participation in, 2021 and normative data. Norm data are derived from the Dutch National Institute of statistics and refers to 2008 [16] Categories are based on normative data derived from the Dutch Institute of Statistics (CBS, 2008). Extensive = 5 or more hours of sport per week, little/moderate = 1–4 h sport per week, little/none = 1 or less hours sport per week.

**Fig. 2:**
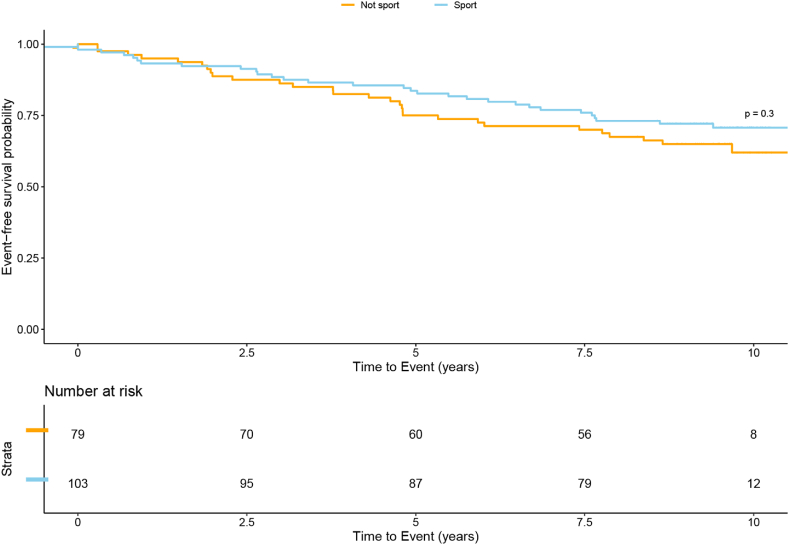
Event-free survival between 2011 and 2021 in sporters and non-sporters 2011 (n = 184) Events were defined as death, symptomatic arrhythmia, re-intervention, stroke, PM or ICD implantation and heart failure.

In 2021, 204 patients (mean age 50 ± 5 years, 46% female) filled out the sports questionnaire of whom 100 (49%) participated in sports. The median follow-up time after surgery was 45.2 years [43.0; 47.2], with a maximum follow-up of 53.7 years post-surgery. Biographical characteristics of patients who participated in the study in 2021 are shown in Table 1. Table 2S in the supplementary material shows biographical characteristics per diagnosis. No significant difference was found between participants in the study (n = 204) and non-participants (n = 139) in terms of diagnosis, sex, and age. Patients with CHD who participated in sports performed better at the exercise test, showing higher exercise capacity (% of predicted norm for healthy peers) (104.1 ± 22.2 vs 88.4 ± 21.1, p < 0.001) and higher VO2 max (97.2 ± 24.4 vs 86.4 ± 21.5, p = 0.003) compared to those who were not practicing any sport. In addition, they showed a lower average heart rate (beats per minute (bpm)) (71 [67–76] vs 77 [69–82], p < 0.001). Type of sports in which the patients participated in 2021 are depicted in Fig. 1S (supplementary material), whereas Fig. 2S (supplementary material) shows specific sports. In 2021, 27.3% of patients were involved in high intensity power sports. Fig. 3S (supplementary material) shows the changes in sports participation from 2011 to 2021. Frequency of sport participation in 2011 and 2021 compared to the Dutch general population is shown in Fig. 1. Overall, in 2021, patients with CHD participated in sports significantly less often than their peers of the same sex and comparable age (data of 2008 were used as no more recent data were available) (male p = 0.03; female: p = 0.03) [16]. No significant difference in hours spent sporting was found over time. Furthermore, patients who did not sport in 2011 but were engaged in sport in 2021 showed a reduction in heart rate (2011: 72 [66–81] bpm vs 2021: 70 [64–76] bpm, p = 0.017) and improvement of exercise capacity (2011: 90 ± 13% vs 2021 97 ± 21%, p = 0.026) in 2021 compared to 2011 (Table 3S).

**Table 1:** Biographical characteristics.

				Congenital heart diseases classification					
	Total			Simple CHD			Moderate/complex CHD		
	**No sport**	**Sport**	p	**No sport**	**Sport**	**P**^a^	**No sport**	**Sport**	**p**^b^
	**n=100**	**n** = **104**		**n=65**	**n = 76**		**n = 35**	**n = 28**	
GENERAL CARACHTERISTICS:									
Congenital Heart Disease			0.212						
Simple	65% (65)	73.1% (76)							
Moderate/complex	35% (35)	26.9% (28)							
Female	49% (49)	42.3% (93)	0.337	50.8%(33)	44.7%(34)	0.475	45.7%(16)	35.7%(10)	0.423
Age (years)	50.4 ± 5.03	49.6 ± 5.3	0.249	51.4 ± 4.8	50.2 ± 5.3	0.764	48.6 ± 4.9	48.0 ± 4.8	0.492
Age at first surgery	5.1 [1.1–7.2]	4.7[1.4–7.1]	0.943	5.6 [2.6–8.2]	5.3 [1.9–8.4]	0.946	3.2 [0.8–6.5]	1.9 [0.7–5.1]	0.609
CARDIAC CARACHTERISTICS:									
Systemic function:			0.887			0.592			0.868
Good	64.8% (59)	65.3% (62)		84.5%(49)	78.6% (55)		30.3% (10)	28.0% (7)	
Reasonable	23.1% (21)	24.2% (23)		12.1% (7)	18.6% (13)		42.4% (14)	40.0% (10)	
Moderate	11.0% (10)	8.4% (8)		3.4% (2)	2.9% (2)		24.2%(8)	24.0%(6)	
Bad	1.1% (1)	2.1% (2)		–	–		30.% (1)	8.0%(2)	
Cardiac medications	47.6% (46)	34.0% (34)	0.048	37.7%(23)	27.8%(20)	0.223	65.7%(23)	50.0% (14)	0.208
Smoking	18.1% (15)	9.2% (9)	0.079	21.1% (12)	11.3% (8)	0.130	11.5% (3)	3.7% (1)	0.280
CPET (% of the norm)	88.4 ± 21.1	104.1 ± 22.2	**<0.001**	90.9 ± 22.8	107.2 ± 20.5	<0.001	83.3 ± 16.4	95.3 ± 24.5	0.019
VO2 max (% of the norm)	86.4 ± 21.5	97.2 ± 24.4	**0.003**	91.6 ± 21.6	100.7 ± 21.6	0.024	73.7 ± 15.3	87.0 ± 29.4	0.067
NYHA class 1	80.7% (71)	90.1% (91)	0.065	85.0% (51)	94.6%(70)	0.048	71.4%(20)	77.8%(21)	0.589
BMI	26.5 [24.0–28.3]	25.3 [23.0–28.1]	0.081	26.58 [24.5–28.1]	25.7 [23.0–29.0]	0.402	26.4 [23.1–29.3]	24.4 [22.8–25.9]	0.106
Diabetes	3.7% (3)	5.2% (5)	0.639	1.8%(1)	4.3%(3)	0.413	8.0%(2)	7.4%(2)	0.936
Hypercholesterolemia	8.4% (7)	8.4% (7)	0.991	7.0%(4)	7.1%(5)	0.989	11.5%(3)	12.0%(3)	0.998
ECG (synus rhytme)	85.2% (82)	84.5% (87)	0.851	85.7%(54)	85.3%(64)	0.367	84.8%(28)	82.1%(23)	0.960
HOLTER DATA									
Avarage bpm	77 [69–82]	71 [67–76]	**<0.001**	76 [69–83]	72 [68–77]	0.005	78 [67–80]	70. [65–76]	0.012
SVT>10	17.6% (16)	8.1% (8)	0.049	16.7% (10)	8.2% (6)	0.136	19.4% (6)	7.7%(2)	0.207
VT (3-10)	9.4% (8)	9.8% (9)	0.933	7.3% (4)	10.4% (7)	0.542	13.4%(4)	8.0%(2)	0.528
PVC >10 complexes	64.8% (59)	67.7% (67)	0.679	58.3%(35)	57.5%(42)	0.926	77.4%(24)	96.2%(25)	0.031
LAS	80 [75–90]	80 [75–90]	0.208	80 [75–90]	80 [75–90]	0.425	80 [75–85]	80 [80–90]	0.312

CPET= Cardio-pulmonary Exercise Test, NYHA=New York Heart Association, BMI= Body Mass Index, SVT = Supraventricular tachycardia, VT=Ventricular tachycardia, PVC = premature ventricular complex, LAS=Linear analog scale Qualitative assessed by the cardiologist according to the current guidelines [38].

a p for simple CHD no sport vs sport.

b p for moderate/complex CHD no sport vs sport.

### 3.2 Quality of life

Table 2 and Fig. 4S (supplementary material) show the scores on the SF-36 of patients with CHD. Of this group, the patients engaged in sports, scored better on all the domains of the SF-36 versus the norm, except for general health (GH) which was similar to the norm. On the contrary, patients with CHD who did not participate in sports, scored significantly worse than the norm in two domains: vitality (VT) (62.0 ± 23.5 vs 68.6 ± 19.3, p = 0.006) and GH (58.9 ± 24.8 vs 70.7 ± 20.7, p < 0.001). Generally, patients who were engaged in sports showed higher scores (better) in 5 domains compared to those who did not sport: physical functioning (PF), role physical (RP), VT, social functioning (SF) and GH.

**Table 2:** SF-36 scores per non sporters and sporters and norm data. Data are presented as mean ± standard deviation. Norm of the general Dutch population is presented [18].

	Norm	Congenital heart disease							
		No sport	Sport	No sport vs Norm		Sport vs Norm		No sport vs sport	
	n = 1742	n = 99	n = 104	t	p	t	p	t	p
**Physical functioning (PF)**	83.0 ± 22.8	81.6 ± 22.1	90.8 ± 17.3	−0.65	0.519	**4.58**	**<0.001**	**−3.32**	**0.001**
**Role Physical (RP)**	76.4 ± 36.3	75.8 ± 40.2	88.2 ± 27.9	−0.16	0.874	**4.33**	**<0.001**	**−2.58**	**0.011**
**Role Emotional (RE)**	82.3 ± 32.9	86.2 ± 30.9	90.1 ± 25.8	1.26	0.212	**3.07**	**0.003**	−0.97	0.333
**Vitality (VT)**	68.6 ± 19.3	62.0 ± 23.5	74.0 ± 18.6	**−2.81**	**0.006**	**2.95**	**0.004**	**−4.05**	**<0.001**
**Mental Health (MH)**	76.8 ± 17.4	80.5 ± 14.4	82.5 ± 14.2	2.57	0.012	**4.12**	**<0.001**	−1.00	0.317
**Social Functioning (SF)**	84.0 ± 22.4	84.8 ± 21.4	91.5 ± 17.7	0.39	0.694	**4.30**	**<0.001**	**−2.41**	**0.017**
**General Health (GH)**	70.7 ± 20.7	58.9 ± 24.8	71.2 ± 21.9	**−4.74**	**<0.001**	0.26	0.798	**−3.77**	**<0.001**
**Bodily Pain (BP)**	74.9 ± 23.4	79.0 ± 23.0	84.0 ± 19.5	1.75	0.083	**4.76**	**<0.001**	−1.69	0.093

### 3.3 Events and mortality

Of the cohort that participated in 2011, 10 patients died in the time between the two follow-ups (10 years). Characteristics of these patients are reported in Table 4S (supplementary material). Of them, 5 were practicing little/moderate sports (1–4 h per week) and one practiced sports extensively (5 or more hours per week). In total 5 patients died due to cardiac causes. Heart failure was the cause of death in 3 patients after Mustard palliation of TGA. In addition, 2 sudden deaths occurred: one in a patient with a VSD and the other in a patient with ToF. The 5 other deaths were non-cardiac. Between 2011 and 2021, cardiac events occurred in 28.2% of patients. No difference was found between patients participating in sports versus patients not practicing sports per diagnostic group for any of the events (Table 5S A-supplementary material). However, patients who had at least an event in their lives were less often participating in sports (Table 5S, B). Fig. 2 shows percentages of event-free survival of the consecutive patients per sports participation in 2011. No significant difference was observed between sporters and non-sporters in terms of event-free survival (p = 0.3).

### 3.4 Lifestyle

Data regarding lifestyle habits in our longitudinal cohort study was assessed for the first time in 2021. Results are shown in Table 3. Compared to the general Dutch population, patients with CHD were smoking significantly less often (10.3% vs 21.1%, p = 0.036). Patients with moderate/severe CHD drank significantly less often more than 6 units of alcohol (60.5% vs 51.0%, p = 0.024) compared to those with a mild defect. For most parameters, no differences were observed between CHD patients and the general population.

**Table 3:** Lifestyle of patients with CHD per diagnosis.

				**Congenital heart diseases classification**		
	**Total**	**Norm**	**p**	**Mild CHD**	**Moderate/severe CHD**	**p**
	**n = 203**			**n = 140**	**n = 63**	
Do you drink alcohol?						
Yes	72.4% (147)	76.7%	0.485	74.3% (104)	68.3% (43)	0.374
How often do you drink alcohol?						0.067
Never	27.5% (56)	–	–	25.7% (36)	31.7% (20)	
Once a month or less	20.1% (41)	–	–	22.9% (32)	14.3% (9)	
2 to 4 times per month	26.1% (53)	–	–	29.3% (41)	19.0% (12)	
2 to 3 times per week	18.6% (38)	–	–	14.3% (20)	18.6% (18)	
> 4 times per week	7.4% (15)	–	–	7.9% (11)	6.3% (4)	
Average unit of alcohol consumed when drinking:			–			0.785
1 to 2	83.7% (123)	–	–	84.8% (88)	81.4% (35)	
3 to 4	15.0% (22)	–	–	14.4% (15)	16.3% (7)	
5 or more	1.4% (2)	–	–	1.0% (1)	2.3% (1)	
How often do you drink more than 6 units of alcohol?						**0.024**
Never	53.7% (79)	–	–	51.0% (53)	60.5% (26)	
Less than once a month	36.1% (53)	–	–	41.3% (43)	23.3% (10)	
Monthly	8.2% (12)	–	–	4.8% (5)	16.3% (7)	
Weekly	2.0% (3)	–		2.9% (3)	–	
Smokers	10.30% (21)	21.1%	**0.036**	10.7% (15)	9.5% (6)	0.768
Drug use in the last year:						
Sleeping or calming medications^b^	9.4% (19)	10.2%	0.849	8.6% (12)	11.1% (7)	0.565
Illegal drug use^a^	2.5% (5)	7.7%	0.095	2.1% (3)	3.2% (2)	0.668
At least once a year to the dentist	92.2% (188)	84.0%	0.073	90.8% (128)	95.2% (60)	0.319
Do you brush your teeth every day?						0.871
No	1.0% (2)	–	–	4.3 % (5)	4.4% (3)	
Yes	32.0% (57)	–	–	95.7% (135)	95.2% (60)	
Do you eat breakfast every day?	81.8% (166)	81.0%	0.884	77.9% (109)	90.5% (57)	0.060
Do you eat fruit every day?	41.4% (84)	–	–	42.1% (59)	39.7% (25)	0.742
Do you eat vegetables every day?	36.5% (74)	–	–	35.7% (50)	38.1% (24)	0.744
How often do you take unhealthy snacks daily?						0.485
Once or less	43.1% (87)	–	–	45.7% (64)	37.1% (23)	
Twice	39.6% (80)	–	–	37.1% (52)	45.2% (28)	
3 or more times	17.4% (35)	–	–	17.1% (24)	17.7% (11)	

a Marijuana, psylocibium, cocaine, ecstasy, speed, or other illicit drugs.

b Benzodiazepine, sedatives, tranquilizers.

## 4 Discussion

In this study of adults, surgically treated for CHD at young age, sports participation was found in half. Interestingly, even if this study was conducted in the Covid-19 pandemic, the percentage of patients who were physically active did not change significantly since 2011. In contrast, the literature regarding physical activity and exercise in adults with CHD showed a wide range of results with normal to limited physical activity in this group of patients [[20], [21], [22]].

Our findings showed that, compared to the general Dutch population, CHD patients, both female and male, dedicate less hours per week to sport activities. Moreover, they are underrepresented in the extensive sports category. A recent study focusing on children and adolescences with CHD showed that 52% of them participated in competitive sports and 23% in recreational ones. Even though good participation was found, this was still lower than the national mean [20]. Differences between adolescents and adults of middle age with CHD are presumably related to different recommendations they received from their physician during childhood. Twenty to thirty years ago physicians used to be more restrictive in fear of severe complications, while nowadays there is more confidence that sports participation is possible and even beneficial also for cardiac patients.

In our study, sports participation was shown to have a positive association with exercise capacity and VO_2_ max. In addition, we found lower heart rate in those who were sporting. This effect of sports on participants was also confirmed when we delved into patients who initiated sporting on 2021. In fact, those patients showed a lower heart rate and better exercise capacity in 2021 compared to 2011. Higher heart rate has shown to be related to higher cardiovascular risk, such as major vascular events, myocardial infarction, stroke, and congestive heart failure [23]. Furthermore, a recent study showed that higher heart rate was associated with lower survival and heart-failure free survival in adults with CHD [24]. Finally, no higher incidence of major cardiac events was observed in patients who were participating in compared to those who were not participating in sports, but as the numbers are limited no firm conclusions can be drawn and further larger trials, preferably randomised controlled trials are warranted. Of course the numbers are still relatively small and indeed larger prospective studies must be awaited, but for now these outcomes are reassuring.

Focusing on quality of life, our earlier investigations have highlighted favourable outcomes among patients with CHD compared to their peers [[25], [26], [27], [28]]. Consistent with these findings, our current study reaffirms this trend. However, our analysis showed that patients with CHD who participate in sports had significantly better quality of life on all domains than the norm, except for the general health perception, which even though better than the norm, the difference was not significant. However, worse scores were found for those who did not participate in sports. Exercise was already found to have a positive effect on psychopathology in this cohort of patients [25,29]. In fact, it was already previously shown that patients engaged in sports have a better mental well-being and quality of life [30,31]. However, we have to take into account that the role of sport in this cohort of patients can be bi-directional. On one hand physical exercise has a well-known positive impact on mental and physical health, but on the other hand, participating in sports may represent that part of the cohort that already has a better physical health and, therefore, is able and willing to participate in sports. Indeed, previous studies have demonstrated that barriers to sports participation can arise from various factors, including socioeconomic status and disabilities [32,33]. Importantly, our findings showed that patients who have experienced major cardiac event in their life are less likely to participate in sports. This reluctance may result from negative advice from clinicians or simply from patients' fear. Overall, considering the benefits of exercise and physical activity, all CHD patients should be encouraged to participate in sports and to be physically active. However, it is fundamental to adjust recommendations on sports activity and intensity per individual. Whereas some patients will be able and may be allowed to participate in all kinds of sports, for more complex diagnoses, limiting exercise to low or medium intensity may be necessary. In 2020, the ESC issued guidelines on sports participation, also including adults with CHD, advising a 5-step approach to identify patients who can sport and those who should limit their sports activity [4]. It might be that these guidelines can be even more liberal in the future.

When focusing on lifestyle, we noticed no significant difference between our CHD patients and the general population, except the positive finding that patients with CHD appeared to smoke less often. This is not a coincidental finding, because other research groups made equal observations [34,35] Possibly, CHD patients are more aware of their health and try to live healthier or are made more aware of the risks of smoking by their cardiologist than healthy individuals [[34], [35], [36]]. Important geographic differences in lifestyle have been reported for CHD subgroups in literature [37]. These are likely to be linked to differences in terms of lifestyles between different countries. Specifically, higher percentages of CHD patients participating in sports were shown in Western and Northern Europe. However, those countries reported higher percentages of adults with CHD who binge drink [37]. In accordance with our study, patients with moderate/severe lesions seem to drink less alcohol than those with mild CHD. This may be explained as CHD patients with moderate/severe defect are more careful in terms of health choices due to their more severe condition. Further research is clearly warranted to illuminate specific motivations for this behaviour.

### 4.1 Strengths and limitations

To our knowledge no other study focused specifically on sports participation in middle aged adults with CHD. We report a follow-up study on the same group of patients previously described a decade ago by Opic et al. [6]. However, it is important to notice that the sample is relatively small, especially for patients with moderate/severe CHD, and therefore no firm conclusion can be drawn. Furthermore, the study had a relatively low participation rate of 59%, and therefore there is a clear need for larger prospective studies, preferably a randomised controlled trial. In addition, we focused on 5 CHD diagnoses, therefore these findings may not be generalized to other diagnostic groups. Specifically, we focused on two moderate/complex CHD diagnoses, therefore generalizability to more complex CHD cases might be limited. Especially patients with a Fontan circulation have a complete different physiological situation and therefore these results cannot be extrapolated to Fontan(s) or other diagnostic groups. In addition, it should be noticed that the study was conducted during the Covid-19 pandemic, therefore sport participation could have been limited by the restrictive measures of that time. However, no significant difference was shown in term of sport participation between the current and the previous investigation in the same cohort. Furthermore, data regarding sport participation were compared with data of the Dutch population of 2008, as no more recent ones were available [16]. Lastly, sports participation was assessed with a self-reported questionnaire and answers may not reflect reality.

## 5 Conclusions

Adults operated for CHD showed lower sports participation than the general population. However, CHD adults who participated in sports had generally a better physical, specifically better exercise capacity and lower heart rate, and better mental health. Sports participation should be encouraged in adults with CHD taking the individual situation into account. Finally, no differences in lifestyle were found between CHD patients and the normal Dutch population, only smoking was less often reported in the CHD group.

## Funding

This work was supported by the “Thorax Foundation”, “Stichting ‘t Trekpaert”, “De Hoop Leven” and “Stichting Pieter Bastiaan”.

## CRediT authorship contribution statement

**C. Pelosi:** Writing – original draft, Visualization, Methodology, Formal analysis, Data curation. **R.M. Kauling:** Writing – review & editing, Methodology, Investigation, Data curation, Conceptualization. **J.A.A.E. Cuypers:** Writing – review & editing, Investigation, Conceptualization. **E.M.W.J. Utens:** Writing – review & editing. **A.E. van den Bosch:** Writing – review & editing, Investigation, Conceptualization. **W.A. Helbing:** Writing – review & editing. **J.S. Legerstee:** Writing – review & editing, Supervision, Conceptualization. **J.W. Roos-Hesselink:** Writing – review & editing, Supervision, Project administration, Methodology, Funding acquisition, Conceptualization.

## Declaration of competing interest

The authors declare the following financial interests/personal relationships which may be considered as potential competing interests:Associate Editor of International Journal of Cardiology: Congenital Heart Disease - J.W. Roos-Hesselink. All other authors declare that they have no known competing financial interests or personal relationships that could have appeared to influence the work reported in this paper.
