# Supplementary material for: Sports participation and lifestyle in middle-aged adults with congenital heart disease
Source: Int J Cardiol Congenit Heart Dis. 2024 Apr 5;17:100512. doi: 10.1016/j.ijcchd.2024.100512 (PMC11657609; doi:10.1016/j.ijcchd.2024.100512)
Supplement: Multimedia component 1 [file mmc1.docx]

**Supplementary material:**

**Figure 1S.** Sport participation of the 2021 cohort according to the sport classification of the ESC [1]


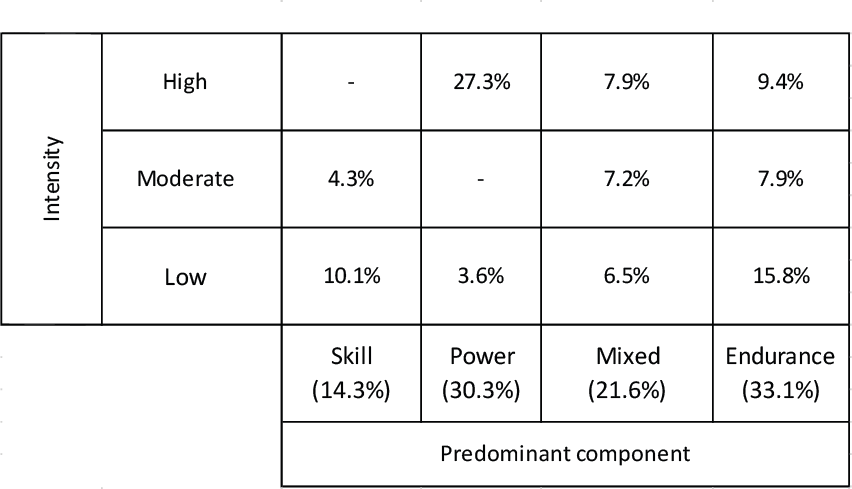


**Skill sports (golf, table tennis, sailing):** achievements depends on technical/bodily skills, without cardiac remodelling.

**Power sports (high-static exercise):** achievements depends on explosive muscle power.

**Mixed sports (ball and team sports)** alternates dynamic and static work and recovery.

**Endurance sports (mid/long distance cycling, running, swimming):** prolonged and intensive high-dynamic and high-static exercise.

**Figure 2S.** Participation per sport type. Number of CHD adults per sport type are shown. (n=35 patients participated

in more than 1 sport)

Other includes athletics, shooting, sailing, handball, honkball, controlled exercise, sports with dogs, dancing etc.

**Figure 3S.** Sport participation in the 2 follow-up times of consecutive patients (n=174).


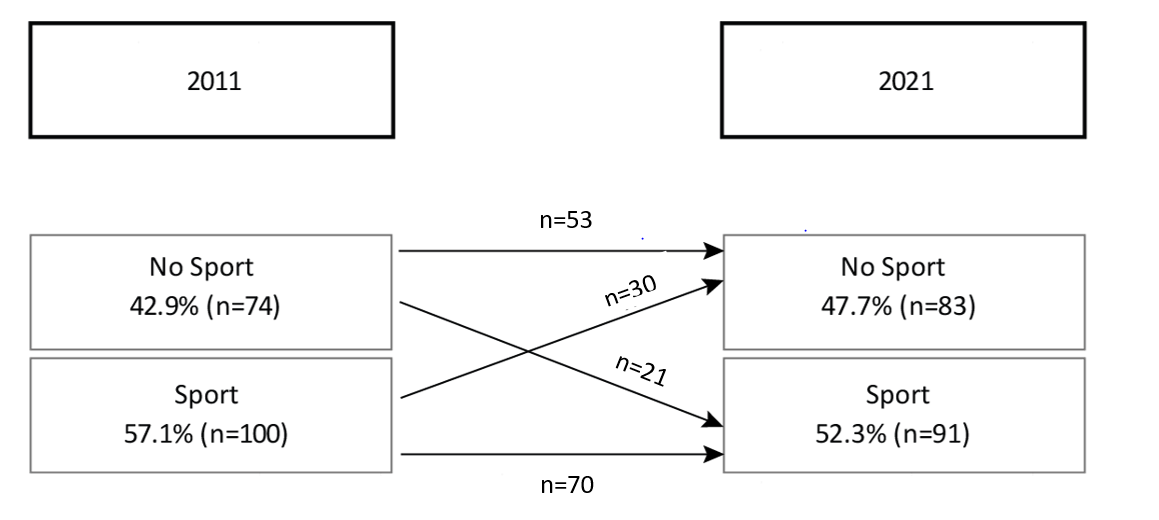


**Figure 4S.** SF-36 scores per no-sporters and sporters and norm data. Data are presented as mean ± standard deviation. Norm of the general Dutch population is presented. [1]

**Table 1S.** Patients flowchart

Original cohort of CHD patients operated between 1968-1980

(n=597)

Alive patients and survival status available at the 4^th^ follow-up time (2021)

(n=431)

Invited patients:

(patients who participated to at least two of the previous follow-up times (2001, n=362; 2011, n=245) and were traceable)

(n=343)

Participants:

(n=204)

**Table 2S.** biographical characteristics per diagnostic group

|  | | | **Congenital Heart Disease** | | | | |  |
| --- | --- | --- | --- | --- | --- | --- | --- | --- |
|  | **ASD** | | | **VSD** | **PS** | **ToF** | **TGA** |  |
|  | **n=58** | | | **n=57** | **n=26** | **n=43** | **n=20** |  |
| Female | 58.6% (34) | | | 38.6% (22) | 42.3% (11) | 44.2% (19) | 35.0% (7) |  |
| Age (years) | 52.2 ± 4.7 | | | 49.5 ± 5.2 | 5.0 ± 5.0 | 49.1 ± 5.3 | 46.6 ± 3.1 |  |
| Age at first surgery | 6.3 [5.2-9.5] | | | 4.3 [0.6-6.6] | 4.9 [2.0-6.4] | 3.8 [1.2-6.6] | 0.7 [0.5-2.8] |  |
| Systemic function: |  | | |  |  |  |  |  |
| Good | 90.6% (48) | | | 82.0% (41) | 60.0% (15) | 41.5% (17) | 11.8% (2) |  |
| Reasonable | 6.9% (4) | | | 16.0% (8) | 32.0% (8) | 53.7% (22) | 70.6% (12) |  |
| Moderate | 1.9% (1) | | | 2.0% (1) | 7.7% (2) | 4.9% (2) | 15.0% (3) |  |
| Bad | - | | | - | - | - | - |  |
| Cardiac medications | 27.3% (15) | | | 38.5% (20) | 30.8% (8) | 48.8% (21) | 80.0% (16) |  |
| Smoking | 14.0% (8) | | | 22.4% (11) | 4.5% (1) | 11.4% (4) | - |  |
| CPET (% of the norm) | 105.4 ± 20.6 | | | 94.8 ± 23.3 | 105.0 ± 19.4 | 97.5 ± 20.0 | 73.8 ± 15.7 |  |
| VO2 max (% of the norm) | 104.5 ± 22.4 | | | 89.3 ± 19.5 | 94.2 ± 20.3 | 91.3 ± 22.0 | 61.2 ± 16.0 |  |
| NYHA class I | 89.7% (52) | | | 92.3% (48) | 87.5% (21) | 86.1% (31) | 52.6 % (10) |  |
| BMI | 25.7 [23.2 ± 28.5] | | | 25.9 [23.1 - 27.7] | 26.6 [24.0-30.5] | 25.0 [23.0-28.2] | 24.8 [23.6-26.8] |  |
| Diabetes | 3.5% (2) | | | 4.2% (2) | - | 3.0% (1) | 15.8% (3) |  |
| Hypercholesterolemia | 9.0% (5) | | | 4.2% (2) | 9.1% (2) | 15.2% (5) | 5.8% (1) |  |
| ECG (synus rhytme) | 78.9% (45) | | | 94.5% (52) | 80.8% (21) | 88.1% (37) | 73.7% (14) |  |
| Holter data |  | | |  |  |  |  |  |
| Avarage bpm | 74.0 [69.0-79.0] | | | 74.0 [69.0-80.0] | 71.5 [64.7-82.2] | 76.0 [67.5-79.0] | 68.0 [64.5-78.0] |  |
| SVT>10 | 16.4% (9) | | | 9.4% (5) | 8.0% (2) | 17.5% (7) | 5.9% (1) |  |
| VT (3-10) | 7.5% (4) | | | 8.3% (4) | 14.3% (3) | 10.5% (4) | 11.8% (2) |  |
| PVC > 10 complexes | 60.0% (33) | | | 56.6% (30) | 56.0% (14) | 82.5% (33) | 94.1% (16) |  |
| LAS | | 80.0 [75.0-90.0] | | 80.0 [75.0-90.0] | 85.0 [73.7-90.0] | 80.0 [75.0-90.0] | 80.0 [80.0-90.0] |  |
| ASD= Atrial Septal defect, VSD=Vetricular Septal Defect, PS=Pulmunary Stenosis, ToF=Tetralogy of Fallot, TGA=Transposition of the Great Arteries, CPET= Cardio-pulmonary Exercise Test, NYHA=New York Heart Association, Qualitative assessed by the cardiologist according to the current guidelines [2] | | | | | | | | |

**Table 3S.** Changes in bio-clinical characteristics in patients who did not sport in 2011 and started engaging sport in 2021

|  | **No sport --> Sport (n=21)** | | |
| --- | --- | --- | --- |
|  | **No sport** | **Sport** | **p** |
| Cardiac medications | - | 15% (3) | 0.063 |
| Smoking | 23% (4) | 12% (4) | 0.125 |
| CPET (% of the norm) | 90.02 ± 13.44 | 97.39 ± 20.80 | 0.026 |
| Holter data |  |  |  |
| Average bpm | 72.5 [66.25- 81.50] | 70.0 [64.5-75.75] | 0.017 |
| SVT>10 | 5% (1) | - | 1 |
| VT (3-10) | 11% (2) | 5.5% (1) | 0.5 |
| PVC > 10 complexes | 5% (1) | 55% (11) | 0.219 |
| LAS | 80.0 [75.0-83.75] | 80.0 [7.0-83.75] | 0.191 |
|  |  |  |  |
| SF- 36 |  |  |  |
| Physical functioning (PF) | 90.95 ± 16.70 | 87.14 ± 21.01 | 0.126 |
| Role Physical (RP) | 91.67 ± 24.15 | 82.14 ± 34.60 | 0.187 |
| Role Emotional (RE) | 90.48 ± 26.12 | 92.06 ± 17.96 | 0.815 |
| Vitality (VT) | 70.71 ± 16.90 | 69.28 ± 17.34 | 0.757 |
| Mental Health (MH) | 82.67 ± 17.72 | 81.90 ± 15.93 | 0.873 |
| Social Functioning (SF) | 92.86 ± 17.03 | 89.28 ± 19.50 | 0.444 |
| General Health (GH) | 74.84 ± 17.90 | 68.09 ± 21.65 | 0.195 |
| Bodily Pain (BP) | 82.76 ± 19.04 | 80.48 ± 18.12 | 0.643 |

**Table 4S.** Data on patients that died between 2011 and 2021.

| **Age** | **Gender** | **Diagnosis** | **BMI** | **Sports/week** | **Cause of death** | **Exercise capacity** | **ECG + Pacemaker** | **Systemic ventricular function** | **Systemic AV-valve** | **Pulmonary valve** | **Arrhythmias** | **Smoking** |
| --- | --- | --- | --- | --- | --- | --- | --- | --- | --- | --- | --- | --- |
| 54 | F | ASD | 24 | Little/moderate | ALS | 83% | SR | Good | Light | No regurgitation | No | No |
| 45 | M | VSD | 25 | Little/moderate | Stroke | N/A | SR + RBTB | Good | Light | No regurgitation | No | Yes |
| 40 | F | Fallot | 36 | None | Sudden death | 82% | - | Bad | No regurgitation | No regurgitation | No | Yes |
| 38 | M | TGA | 30 | None | Heart failure | 55% | SR + 1st grade AV block | Bad | Mild regurgitation | Light | No | No |
| 39 | M | TGA | 24 | None | Heart failure | 43% | PM | Bad | Severe regurgitation | No regurgitation | AF/VF(2005) | No |
| 46 | F | TGA | 31 | Little/moderate | Heart failure | 70% | SR + RBTB | Mild dysfunction | Light | Light | AF (2018) | No |
| 47 | F | ASD | 27 | Little/moderate | Brain tumour | 108% | SR | Good | Light | No regurgitation | No | No |
| 35 | M | VSD | 28 | None | Sudden death | 122% | PM | Good | No regurgitation | No regurgitation | No | No |
| 52 | M | PS | 27 | Extensive | Cancer | 117% | SR + RBTB | Good | Mild regurgitation | Light | No | No |
| 40 | M | TGA | 22 | None | ACUP | 68% | SR + 1st grade AV block | Mild dysfunction | Light | No regurgitation | Mobitz 2 | No |

**Table 5S.** events between 2011 and 2021 divided per sport participation in 2011 of patients for continuous patients (n=175) who participated to all 3 FU times (2001, 2011, 2021). Events between 2001 and 2011 are reported in the previous paper. [3]

|  | | | | | | | | |
| --- | --- | --- | --- | --- | --- | --- | --- | --- |
|  |  |  | **Congenital heart diseases classification** | | | | | |
|  | **Total** |  | **Simple CHD** | |  | **Moderate/ complex CHD** | |  |
|  |  |  | **No sport** | **Sport** | **p** | **No sport** | **Sport** | **p** |
|  | **n = 174** |  | **n = 48** | **n = 73** |  | **n = 26** | **n = 27** |  |
| Major events |  |  |  |  |  |  |  |  |
| Overall* | 28.2% (49) |  | 22.9% (11) | 17.8% (13) | 0.491 | 53.8% (14) | 40.7% (11) | 0.339 |
| Arrhythmia | 19.0% (33) |  | 12.5% (6) | 8.2% (6) | 0.445 | 46.2% (12) | 33.3% (9) | 0.340 |
| PM/ICD implantation | 4.6% (8) |  | 2.0% (1) | 2.7% (2) | 0.818 | 11.5% (3) | 7.4% (2) | 0.606 |
| Re-intervention | 11.5% (20) |  | 8.3% (4) | 2.7% (2) | 0.171 | 26.9% (7) | 25.9% (7) | 0.934 |
| Stroke | 4.6% (8) |  | 8.3% (4) | 4.1% (3) | 0.337 | 3.8% (1) | - | 0.229 |
| Heart failure | 3.4% (6) |  | - | - | - | 11.5% (3) | 11.1% (3) | 0.961 |
| *Patients with at least one event Some patients had more than 1 event, therefore the total sum of all the singular events is higher than 59. | | | | | | | | |

**A-**

**B-** Major events in sport and no-sport participant.

|  | **Total** |  | **Sport** | **No sport** |  |
| --- | --- | --- | --- | --- | --- |
|  | **(n=204)** |  | **(n= 104)** | **(n=100)** | **p** |
| **Major events since (since corrective surgery till 2021)** |  |  |  |  | 0.037 |
| **Yes** | 40.7% (83) |  | 33.7% (35) | 48.0% (48) |  |
| **No** | 59.3% (121) |  | 63.0% (69) | 52.0% (52) |  |

**References:**

1. Budts, W., et al., *Recommendations for participation in competitive sport in adolescent and adult athletes with Congenital Heart Disease (CHD): position statement of the Sports Cardiology & Exercise Section of the European Association of Preventive Cardiology (EAPC), the European Society of Cardiology (ESC) Working Group on Adult Congenital Heart Disease and the Sports Cardiology, Physical Activity and Prevention Working Group of the Association for European Paediatric and Congenital Cardiology (AEPC).* Eur Heart J, 2020. **41**(43): p. 4191-4199.
2. Aaronson, N.K., et al., *Translation, validation, and norming of the Dutch language version of the SF-36 Health Survey in community and chronic disease populations.* J Clin Epidemiol, 1998. **51**(11): p. 1055-68.
3. Lang, R.M., et al., *Recommendations for cardiac chamber quantification by echocardiography in adults: an update from the American Society of Echocardiography and the European Association of Cardiovascular Imaging.* Eur Heart J Cardiovasc Imaging, 2015. **16**(3): p. 233-70.
4. Opic, P., et al., *Sports participation in adults with congenital heart disease.* Int J Cardiol, 2015. **187**: p. 175-82.
